# Early sedation use in critically ill mechanically ventilated patients: when less is really more

**DOI:** 10.1186/s13054-014-0600-3

**Published:** 2014-11-10

**Authors:** Christie M Lee, Sangeeta Mehta

**Affiliations:** Department of Medicine and Interdepartmental Division of Critical Care Medicine, Mount Sinai Hospital, University of Toronto, 600 University Avenue, Toronto, ON M5G 1X5 Canada

## Abstract

Over the last 10 years, there has been an explosion of literature surrounding sedation management for critically ill patients. The clinical target has moved away from an unconscious and immobile patient toward a goal of light or no sedation and early mobility. The move away from terms such as ‘sedation’ toward more patient-centered and symptom-based control of pain, anxiety, and agitation makes the management of critically ill patients more individualized and dynamic. Over-sedation has been associated with negative ICU outcomes, including longer durations of mechanical ventilation and lengths of stay, but few studies have been able to associate deep sedation with increased mortality.

## Commentary

In a previous issue of *Critical Care*, Tanaka and colleagues [1] evaluate the association of early sedation and important ICU-related outcomes. These investigators performed a secondary analysis of a multicenter cohort study of 322 mechanically ventilated sedated patients in 45 Brazilian ICUs. They found that early deep sedation, defined by a Glasgow Coma Scale (GCS) score of less than 9 on the second day of mechanical ventilation, was present in 35% of all patients and that, after adjustment for confounders, early deep sedation was independently associated with increased hospital mortality. In unadjusted analyses, patients with a GCS score of less than 9 had higher overall severity of illness on the basis of higher Simplified Acute Physiology Score 3 scores, longer duration of ventilator support, and a higher rate of tracheostomy insertion, compared with those with a GCS score of 9 or more. Exclusive use of fentanyl or dexmedetomidine was also more prevalent in lightly sedated patients compared with deeply sedated patients [1].

The strengths of this study include the multicenter design and broad inclusion criteria, which increase the generalizability of the study results. Few studies have evaluated the first 24- to 48-hour period of mechanical ventilation and sedation. The focus on the early time period adds to the growing knowledge in this area, demonstrating an association between early deep sedation and ICU mortality. The results of this study are similar to those of two other trials, showing that early sedation was an independent predictor of time to extubation, hospital mortality, and 180-day mortality [2,3]. This study has limitations, which were acknowledged by the authors. This is a secondary analysis of an observational trial, and this fact limits our ability to make conclusions, and even though adjustments are made for potential confounders, missed variables such as mechanical ventilation modes and intensity can also contribute to the observed mortality difference between groups. The use of GCS rather than a validated sedation scale such as the Richmond Agitation-Sedation Scale or the Sedation-Agitation Scale [4] limits conclusions of this study, as the GCS has more inconsistent inter-observer reliability, particularly in non-verbal intubated patients, compared with validated sedation scales [5]. The investigators evaluated GCS score on day 2 of mechanical ventilation—thus, we do not know the neurological status of the patient at the time of hospital admission—or on day 1 of mechanical ventilation. This is important because a low GCS score may not be related to sedation, but may be related to poor baseline neurological status, encephalopathy related to critical illness, or drugs administered prior to ICU admission. It is also not known whether sedation protocols were used in the included ICUs, as their use may reduce the likelihood of over-sedation. Finally, the incidence of delirium was not captured during the study. Delirium is an independent predictor of both hospital and 6-month mortality [6,7]. Most delirium within the ICU is hypoactive and, as a result, may not be recognized, even in those patients who are lightly sedated.

Despite these limitations, it is apparent that early deep sedation is prevalent among ICU patients and may contribute to increased mortality. The desire to treat pain, anxiety, and discomfort is natural for all clinicians; however, critical care physicians often struggle to balance an appropriate level of symptom control and the avoidance of over-sedation. Early in the patient’s critical illness, health providers may administer sedatives to enhance patient comfort and safety and reduce the potential for complications. ICU patients may require sedation for invasive procedures or emergent in-hospital transfers for diagnostic tests. To prevent unexpected extubation, to optimize mechanical ventilation in severe acute respiratory distress syndrome, and to alleviate the distress of patients and family members, health-care providers may administer deep sedation early on, without recognition of the potential adverse consequences. Tanaka and colleagues add to our knowledge of the consequences of early over-sedation and underscore the need for research on early sedation practices and ICU outcomes as well as for attention to patient inclusion criteria for future randomized trials. A recent pilot study by Shehabi and colleagues [8] evaluated the feasibility of an early goal-directed sedation strategy with avoidance of early deep sedation in critically ill mechanically ventilated adults. In that study, patients randomly assigned to early goal-directed sedation were more likely to be lightly sedated and had more delirium-free days and less use of physical restraints compared with standard care [8]. The investigators are conducting a definitive multicenter randomized trial, SPICE III (Sedation Practice in Intensive Care Evaluation III) (ClinicalTrials.gov NCT01728558) [9].

In our practice, we focus on patient-centered specific symptom control; we assess and treat pain, anxiety, sleep deprivation, and delirium before considering deeper sedation to control dangerous agitation. Despite this, it is surprising how often during ICU multidisciplinary rounds that we continue to identify over-sedated patients who are labeled as calm and appropriate, despite the use of a validated sedation scale. The determination of proper interpretation and use of ICU sedation scales remains a challenge. In addition, though paramount to our patients’ outcomes, delirium screening, a key component of the ICU neurological exam, is often forgotten. Although the culture is shifting away from the sedate immobile patient, we are still far from the ideal: awake, interactive, and mobile. Culture is often engrained in the past, and to change culture, we must work together to transform practice. It is evident from the studies by Tanaka and colleagues [1] and others [3,8] that sedation depth early in critical illness adversely impacts patient outcomes. We eagerly await the results of the SPICE III randomized controlled trial, but until then we should all strive to avoid early deep sedation in critically ill patients.

## Abbreviations

GCS: Glasgow Coma Scale
SPICE: Sedation Practice in Intensive Care Evaluation
